# Triptans and Lasmiditan Are Safe and Effective in Patients with Migraine Attacks Associated with Neurodevelopmental Disorders: A Single-Center Prospective Clinical Case Series

**DOI:** 10.3390/brainsci16101036

**Published:** 2026-09-27

**Authors:** Yasushi Shibata

**Affiliations:** Department of Neurosurgery/Headache Clinic, Mito Medical Center, University of Tsukuba, Mito Kyodo General Hospital, Mito 310-0015, Ibaraki, Japan; yshibata@md.tsukuba.ac.jp; Tel.: +81-29231-2371

**Keywords:** migraine, triptan, lasmiditan, neurodevelopmental disorder

## Abstract

**Highlights:**

**What are the main findings?**

- Triptans and lasmiditan (50 mg) were effective in patients with migraine and neurodevelopmental disorders, providing pain relief within 2 h in nearly all patients, and 73% and 50% of patients were pain-free for 2 h.
- Adverse event rates of triptans and lasmiditan were very low in these patients.

**What are the implications of the main findings?**

- Triptans and lasmiditan are effective and safe for patients with migraine and neurodevelopmental disorders in this case series.
- These findings should be validated in a large clinical study.

**Abstract:**

**Background:** Triptans and lasmiditan are considered specific acute medications for migraine attacks. Despite the efficacy of these treatments, continuation of these medications is limited because some patients experience adverse effects. A previous study reported a close association between migraine and neurodevelopmental disorders. The present study investigated triptans and lasmiditan in the treatment of consecutive patients with migraine attacks accompanied by neurodevelopmental disorders. **Methods:** The enrolled patients were diagnosed with migraine by a certified headache specialist. Neurodevelopmental disorders were diagnosed by multiple specialists. Triptan and/or lasmiditan tablets were prescribed for all patients. **Results:** Triptans were effective in these patients, providing pain relief within 2 h in all patients, and twelve patients (80%) were pain-free at 2 h. However, throat discomfort was detected in two patients, and it was regarded as a minor adverse event. Lasmiditan (50 mg) was prescribed to 12 patients, and it provided pain relief in 11 patients (92%) at 2 h and rendered 7 patients pain-free (58%) at 2 h. Conversely, mild somnolence and mild dizziness were recorded in two and one patient, respectively (25%). **Conclusions:** In this study, triptans and lasmiditan were effective and safe in the treatment of migraine attacks accompanied by neurodevelopmental disorders. In this study, several different acute treatments were evaluated non-randomly in a small number of patients, making direct comparisons between treatments difficult. The results of our case series should be clarified in future clinical studies with larger patient populations.

## 1. Introduction

Migraine is a highly prevalent neurological disorder [1]. Because of the higher prevalence of migraine in patients of working age, its socioeconomic burden has been large [2]. The systematic analysis of the Global Burden of Disease Study 2016 demonstrated that 3 billion individuals had a migraine or tension-type headache in 2016 and that migraine caused 45.1 million years of life lived with disability [3].

Multiple medications have been used to mitigate acute migraines. Nonsteroidal anti-inflammatory drugs (NSAIDs) have been frequently used to treat acute migraines, but they are not always effective. Long-term NSAID overuse increases the risk of several complications, such as medication overuse headache [4]. Thus, more effective and specific drugs lacking deleterious effects should be used as acute treatments for migraine attacks. For more than 30 years, triptans have been used as specific acute medications for migraine. The drugs are available as subcutaneous injections, tablets, nasal injections, and rapidly dissolving tablets. Although triptans are effective against most migraines, they do not alleviate some migraine attacks, and some patients experience adverse effects [5]. Frequent use of triptans can also cause medication overuse headaches.

Several previous basic and clinical studies reported calcitonin gene-related peptide (CGRP) as one of the major neurotransmitters mediating migraine attacks [6,7]. Thus, anti-CGRP monoclonal and anti-CGRP receptor monoclonal antibodies are clinically available as effective treatments to prevent migraine attacks. These monoclonal antibodies do not penetrate the blood–brain barrier because of their high molecular weights; thus, their main working sites are the trigeminal nerve terminals and trigeminal ganglia. Gepants are small-molecule CGRP receptor antagonists that are available under multiple brands [8,9]. Some gepants are regarded as acute medications, and others are used as preventive treatments for migraine attacks.

Triptans are serotonin (5-HT) 1D (5-HT1D) and 1B (5-HT1B) receptor agonists. The 5-HT1D receptor is located on the nociceptive Aδ-fiber terminals of the trigeminal nerves. Triptans inhibit signal transduction by Aδ-fibers. Conversely, the 5-HT1B receptor is distributed on blood vessels. Thus, triptans have vasoconstrictive effects.

Ditans are selective 5-HT1F receptor agonists. The 5-HT1F receptor exists on both the C-fiber and Aδ-fiber terminals of the trigeminal nerves [10]. Ditans inhibit both the release of CGRP from the C-fiber terminal and signal transduction in Aδ-fibers. Lasmiditan is a newly developed ditan used in the acute treatment of migraine. Lasmiditan has a high rate of adverse effects, including somnolence and dizziness, despite its high efficacy in alleviating migraine attacks [11]. This high rate of adverse effects of lasmiditan is attributed to the fact that it easily enters the central nervous system.

Neurodevelopmental disorders also represent highly prevalent health issues [12]. The American Psychiatric Association has defined the diagnostic criteria and classified these health issues, as published in the 5th edition of the Diagnostic and Statistical Manual of Mental Disorders (DSM-5-TR) [13]. In reference to the DSM-5, neurodevelopmental disorders have been classified into several types, including communication and special learning disorders, intellectual disability, motor disorders, autism spectrum disorder (ASD), and attention-deficit/hyperactivity disorder (ADHD).

Several studies have reported a close association between migraine and neurodevelopmental disorders [14,15,16]. Compared with the findings in healthy subjects, the concentration of 5-HT was lower in the brain and higher in the blood in patients with neurodevelopmental disorders [17,18]. Additionally, patients with neurodevelopmental disorders have been found to exhibit reduced 5-HT metabolism and lower 5-HT receptor expression in the brain [19,20]. These basic findings suggest that 5-HT agonists may exert different therapeutic effects and adverse event profiles in migraine patients with and without neurodevelopmental disorders.

For patients with migraine accompanied by neurodevelopmental disorders, our clinical experiences suggested mild effects associated with 5-HT agonists. However, the clinical effects of triptans and lasmiditan in the treatment of migraine attacks accompanied by neurodevelopmental disorders have not been previously reported. In this study, consecutive patients with migraine accompanied by neurodevelopmental disorders were prospectively treated with triptans and/or lasmiditan, and their efficacy and adverse effects were investigated. This pilot case series aims to test the hypothesis that triptans and lasmiditan are effective and safe for patients with migraine and neurodevelopmental disorders.

## 2. Materials and Methods

In this single-center prospective clinical study, patients were diagnosed with migraine by a certified headache specialist according to the International Classification of Headache 3rd edition [21]. For all patients, brain magnetic resonance imaging was performed, and other organic lesions causing headache were eliminated. Before this study, neurodevelopmental disorders were diagnosed by several specialists, including psychiatrists, pediatric neurologists, and neurologists. Triptans and/or lasmiditan tablets were prescribed for consecutive patients after the diagnosis of migraine and neurodevelopmental disorders. The choice of treatment was determined by the treating physician. Triptans, eletriptan or zolmitriptan was prescribed. However, in this study, lasmiditan was used at a dose of 50 mg for all patients, including children, because the 100 mg dose caused substantial adverse effects in Japanese patients [22]. Concomitant preventive therapies were kept unchanged throughout the observation period.

Using a paper diary, all enrolled patients were instructed to record their headaches, the effects of triptans or lasmiditan, and their adverse effects. In this study, the Japanese version of the paper diary was used. Table 1 presents the English version of the same paper diary. At the next clinical visit, typically occurring 1 month later, the efficacy and adverse effects of the treatments were confirmed, and the patients were interviewed. Based on the information recorded in the paper diary, pain relief and pain-free rate at 2 h of some migraine treatments were evaluated. Moreover, any harmful effects were confirmed in the paper diary and clinical interviews. When a patient used oral medication for multiple migraine attacks, the treatment effect was averaged, and all adverse events were recorded.

Before starting the investigation, this clinical study was approved by the internal review board of the University of Tsukuba’s Mito Medical Center (code No. 24-85). Written informed consent was obtained from all participants and their caregivers.

This case series was conducted using a single-arm design without a control group, and no statistical analysis was performed.

## 3. Results

A total of 21 patients with migraine and neurodevelopmental disorders were screened for study eligibility. Two patients declined to participate, and another two did not return for the follow-up visit. Consequently, 17 patients were included in this case series (Table 2). The ages of these patients ranged from 9 to 39 years. The cohort included eight males and nine females. Migraine attacks associated with and without aura were observed in 5 and 12 patients, respectively. The frequency of migraine attacks was episodic and chronic in eight and nine patients, respectively. Preventive medications for migraine were prescribed to 14 patients, and 2 patients were prescribed both oral medication and anti-CGRP monoclonal antibodies. Regarding neurodevelopmental disorders, seven patients were diagnosed with ASD, two patients were diagnosed with Asperger’s syndrome, and eight patients were diagnosed with ADHD. The summary of the patient characteristics is presented in Table 3. All patients were Japanese, and all patients had the ability to describe their headaches and the efficacy and adverse effects of the medications.

Triptans were prescribed to 15 patients. Triptans provided pain relief at 2 h in all patients, and 12 patients (80%) were pain-free at 2 h (Figure 1). Throat discomfort was recorded as a minor adverse effect of triptans in two patients (13%). Lasmiditan (50 mg) was prescribed to 12 patients. Pain relief at 2 h was achieved in 11 patients (92%), and 7 patients (58%) were pain-free at 2 h (Figure 2). Meanwhile, mild somnolence and mild dizziness were recorded in two and one patient, respectively (25%).

## 4. Discussion

### 4.1. Clinical Problems in the Acute Treatment of Migraine

To treat acute migraine, some specific drugs have been used. Triptans have long been prescribed and are generally considered effective for most patients. They have been recommended as the first choice for treatment for acute migraine attacks in the clinical guidelines issued by the International Headache Society and the American Headache Society [23,24].

Triptans are available in various brands and dosage forms, including subcutaneous injections, hard tablets, nasal injections, and rapidly dissolving tablets [5]. Triptan use has been associated with adverse effects such as throat discomfort. Because triptans are 5-HT1B receptor agonists, they are contraindicated in patients with cardiac or cerebrovascular diseases because of their vasoconstrictive effects [25].

Lasmiditan is a newly marketed ditan, which is considered a class of specific 5-HT1F receptor agonists. The adverse effects of ditans are not worrying because they do not target the 5-HT1B receptor. However, the adverse effects of lasmiditan, including somnolence and dizziness, are frequent and these adverse effects limit the wide-scale clinical use of this treatment. Phase 2/3 clinical trials of lasmiditan reported frequent adverse effects and strong impacts [11,26].

Gepants comprise a new class of oral anti-CGRP receptor antagonists. Ubrogepant and rimegepant are used in the acute treatment of migraine attacks [27,28]. The adverse effect rate of gepants is lower than those of triptans and lasmiditan. The clinical efficacy of gepants for the treatment of migraine attacks was comparable to that of triptans and lasmiditan [29]. However, gepants have longer half-lives in the blood than triptans and lasmiditan. Thus, they can suppress migraines for a longer period. The high costs of gepants compared with those of triptans and lasmiditan prevent their wide-scale use in the treatment of migraine attacks. CGRP-targeted medications have also been used for the prevention of migraine attacks. Numerous clinical and neurophysiological effects of these therapies have been reported in the literature [30,31].

### 4.2. The Association Between Migraine and Neurodevelopmental Disorders

The clinical relationship between migraine and neurodevelopmental disorders has been investigated. A US National Health Survey and large academic health system electronic health records indicated that migraine occurs at higher rates in individuals with autism [15]. The global prevalence of ADHD is high in males at approximately 5% [12]. Both migraine attacks and tension-type headache have strong associations with ADHD [12]. Clinical evidence has suggested several common pathological mechanisms leading to both migraine attacks and neurodevelopmental disorders, although the mechanisms have not been clearly identified.

5-HT is a key neurotransmitter targeted in the treatment of both migraine attacks and neurodevelopmental disorders. Triptans and lasmiditan are 5-HT receptor agonists used in the acute treatment of migraine attacks. Frequently used treatments for neurodevelopmental disorders include 5-HT reuptake inhibitors (SSRIs) and 5-HT noradrenaline reuptake inhibitors. Combined use of triptans and SSRIs has been found by the US Food and Drug Administration to cause 5-HT syndrome. However, several studies have supported the safe concomitant use of triptans and SSRIs [32,33].

### 4.3. 5-HT Abnormalities in Neurodevelopmental Disorders

There is an increase in the 5-HT concentration in the blood during the fetal period, but the number of 5-HT receptors declines in the brain during normal development. After birth, the number of 5-HT receptors in the brain increases until 2 years or age, after which it decreases [34]. In ASD and during the fetal period, the 5-HT concentration in the blood does not increase. In addition, the number of 5-HT synapses does not decrease after birth. Patients with ASD are characterized as having a weak ability to produce 5-HT [35] but high 5-HT concentrations in the blood and platelets [36], in addition to low 5-HT2A receptor binding in the cerebrum and platelets [37]. Patients with ASD have been reported to have low 5-HT concentrations in the brain and cerebrospinal fluid but high concentrations in platelets [17,18]. Previous imaging studies in such patients, including positron emission tomography and single-photon emission computed tomography, revealed a low density of 5-HT transporters in the frontal lobe and basal ganglia [20,34,36]. These findings might support the hypothesis that 5-HT agonists may have mild clinical effects and a low rate of adverse effects in patients with neurodevelopmental disorders.

### 4.4. Clinical Implications of the Results of This Study

For patients with migraine, triptans have high efficacy and low rates of adverse effects. Because this case series does not include a control group, our results were indirectly compared with findings from clinical trials of triptans and lasmiditan conducted in Japanese populations (Figure 1 and Figure 2). Although both our patients and those in previous trials were Japanese individuals with migraine, the patient populations and study conditions differed. Therefore, this indirect comparison should be interpreted with caution. For Japanese patients with migraine attacks, a phase 3 study of eletriptan (20 mg) revealed a high pain-relief rate of 64% at 2 h post-dose; however, the observed pain-free rate at 2 h post-dose was 24% [37]. Meanwhile, a phase 3 study of zolmitriptan (2.5 mg) in Japanese patients with migraine attacks recorded a similarly high pain-relief rate of 56% at 2 h post-dose and a 2-h pain-free rate of 19% [38]. In prior research, the adverse event rate was 16% for eletriptan (20 mg) and 19% for zolmitriptan (2.5 mg; Figure 1), in line with the rate of 18% in the current study. In the current population of patients with migraine attacks accompanied by neurodevelopmental disorders, the pain-relief rate and pain-free rate at 2 h post-dose for triptans were 100% and 73%, respectively. In a phase 3 clinical trial of Japanese patients, the recorded pain-relief rates and pain-free rates at 2 h post-dose for eletriptan and zolmitriptan were approximately 60% and 20%, respectively [37,38]. Thus, the pain-relief and pain-free rates were higher in the current population of patients with migraine attacks accompanied by neurodevelopmental disorders. In a phase 2/3 study, lasmiditan exhibited strong efficacy in the treatment of the Japanese patients with migraine [22]. For lasmiditan (100 mg), the rate of adverse events was high (81%), but the pain-relief rate at 2 h post-dose was 80%, and the 2-h pain-free rate was 32%. At a lasmiditan dose of 50 mg, the observed pain-relief rate at 2 h post-dose was 68%, the pain-free rate was 24%, and the rate of adverse events was 65% [22]. The effects of lasmiditan as an additional treatment for triptan nonresponders were reported in a previous study [39]. Treatment with triptans followed by lasmiditan (50 mg), used as a preplanned step of care, led to a pain-relief rate of 86% and a pain-free rate of 57%, surpassing the rates in the phase 2/3 clinical trial [22]. The recorded rates of adverse events were similar to those of lasmiditan 50 mg (Figure 2). In the present study, the pain-relief rate of lasmiditan 50 mg was 90% at 2 h post-dose, and the pain-free rate was 50%. These results may indicate better efficacy than was observed in previous clinical trials in Japan [22] and similar efficacy to our previously conducted clinical trial, which used lasmiditan as an additional treatment for triptan nonresponders [39]. In the current population study, the adverse event rate of lasmiditan was 25%.

For patients with migraine attacks accompanied by neurodevelopmental disorders, the current results revealed the high efficacy and low adverse event rates of triptans and lasmiditan. Meanwhile, a high rate of adverse events has represented a major clinical problem for lasmiditan. Health claims data had clearly demonstrated early discontinuation of lasmiditan treatment in Japanese patients with migraine attacks [40]. Adverse events arising from the central nervous system were considered the cause of treatment discontinuation [41]. In the present study population, lasmiditan was associated with infrequent adverse effects. This result might indicate that the central nervous system of patients with neurodevelopmental disorders may be resistant to the deleterious effects of 5-HT receptor agonists, mainly triptans and lasmiditan. The observed low rate of adverse effects was suggestive of strong clinical efficacy, low discontinuation rates, and longer treatment continuation.

The mean number of monthly migraine days in the phase 3 study of zolmitriptan (2.5 mg) in Japanese patients with migraine was 3 [38]. The number of monthly migraine days in the phase 2/3 study of lasmiditan in Japanese patients with migraine ranged from 3 to 8 [22]. Only 37.5% of the patients in this phase 2/3 study of lasmiditan were prescribed preventive medications for migraine attacks. The current case series included 53% of patients with chronic migraine, and 82% of patients were prescribed preventive medications for migraine attacks. Therefore, the current study population was a more severe and difficult-to-treat cohort.

Triptans and lasmiditan are the most commonly used specific drugs for the acute treatment of migraine attacks. However, some patients did not experience sufficient efficacy, and others displayed adverse effects. For patients with migraine attacks accompanied by neurodevelopmental disorders, the current study supports a strong efficacy and low adverse event rates for triptans and lasmiditan. Thus, triptans and lasmiditan could be prescribed for patients with migraine attacks accompanied by neurodevelopmental disorders. The recorded clinical efficacy and adverse effects of lasmiditan were dose-dependent [42]. The dose of lasmiditan could be increased to achieve sufficient efficacy for patients with neurodevelopmental disorders, given its low rate of adverse effects. The clinical trial of lasmiditan included only adult participants [22]. Therefore, prescribing lasmiditan for pediatric patients requires caution; however, it is not contraindicated. In this cohort, patients aged 12 years and older received lasmiditan 50 mg, and adverse events were less frequent than those reported in adult clinical trials.

Several previous studies examined the 5-HT concentration in platelets and the 5-HT receptor density in patients with medication overuse headache [43,44]. The 5-HT concentrations in the platelets of patients with migraine attacks were the same as those in the healthy controls. However, patients with medication overuse headache displayed remarkably lower 5-HT concentrations. In patients with medication overuse headache, the 5-HT receptor density was the same as that in the healthy controls, but patients with migraine exhibited a considerably lower density. This study additionally demonstrated that the 5-HT receptor density was lower in the evolution to medication overuse headache. When the migraine progressed to medication overuse headache, 5-HT concentrations in platelets decreased. The pathophysiology of migraine and therapeutic medications could be considered the causes of these observed changes. In contrast to the trends in medication overuse headache, patients with neurodevelopmental disorders displayed a high 5-HT concentration in platelets and a low 5-HT receptor density. These findings support the possibility that patients with neurodevelopmental disorders are resistant to medication overuse headaches. Accordingly, more prospective clinical research is needed to answer this question.

### 4.5. Limitation of This Study

Despite its prospective design, this case series had several major limitations. Because this clinical case series was open-label, the potential influence of the placebo effect must be taken into account when comparing our findings with those from double-blind phase 3 trials. The sample size was small, the age range of participants was heterogeneous, and patients received different acute treatments. Moreover, this case series was conducted at single center and used a single-arm design without a control group, precluding any statistical comparisons. Future large-scale clinical studies should include two well-matched patient groups: one with migraine and neurodevelopmental disorders and another with migraine but without neurodevelopmental disorders. The two groups should control for key variables such as age, sex, ethnicity, body weight, migraine subtype (with or without aura), migraine frequency, concomitant medications, comorbidities, and living and work environments. Because this case series reflects the experience of a single-institution and all patients were Japanese, the generalizability of these findings to other ethnic groups or clinical settings is limited. The effects of the medications were subjectively evaluated by the patients themselves. A paper diary was used to record migraine attacks and the effects of the medications. Some patients might not have been able to record these results immediately after migraine attacks. Thus, the possibility of recall bias cannot be excluded. The use of an e-diary or an automatic recording system might help eliminate recall bias in future studies. The therapeutic and adverse effects of the treatments were only evaluated at 2 h post-dose. Therefore, their long-term impacts were not determined. Frequent medication use might lead to different therapeutic and adverse effects. A clinical study of lasmiditan recorded a gradual decrease in delirious effects after continuous intake [45]. Accordingly, the adverse effects of lasmiditan could decrease following continuous intake. In the current case series, tablets of eletriptan, zolmitriptan, or lasmiditan (50 mg) were only prescribed as acute treatments for migraine attacks. Several triptans have variable dosage forms, such as nasal and subcutaneous injections. The effects of other drug brands, forms, or doses including lasmiditan 100 mg were not evaluated. In a future study, appropriate selection of triptan brands, forms, and doses should be investigated in large patient populations. Our case series included some pediatric patients, and their clinical results were almost identical to those of adult patients. Differences between adult and pediatric patients and appropriate medications should be investigated in future studies. Adverse events were evaluated solely through patient interviews and paper diaries. Systematic assessments of neurological, behavioral, cognitive, or sensory adverse effects may be valuable for better understanding adverse events in patients with neurodevelopmental disorders. Our case series demonstrated real-world experience of the efficacy and safety of triptan and lasmiditan in patients with migraine and neurodevelopmental disorders.

## 5. Conclusions

In our case series, triptans and lasmiditan (50 mg) exhibited efficacy and safety in the treatment of migraine attacks accompanied by neurodevelopmental disorders. The results of our case series should be clarified in future clinical studies with larger patient populations.

## Figures and Tables

**Figure 1:**
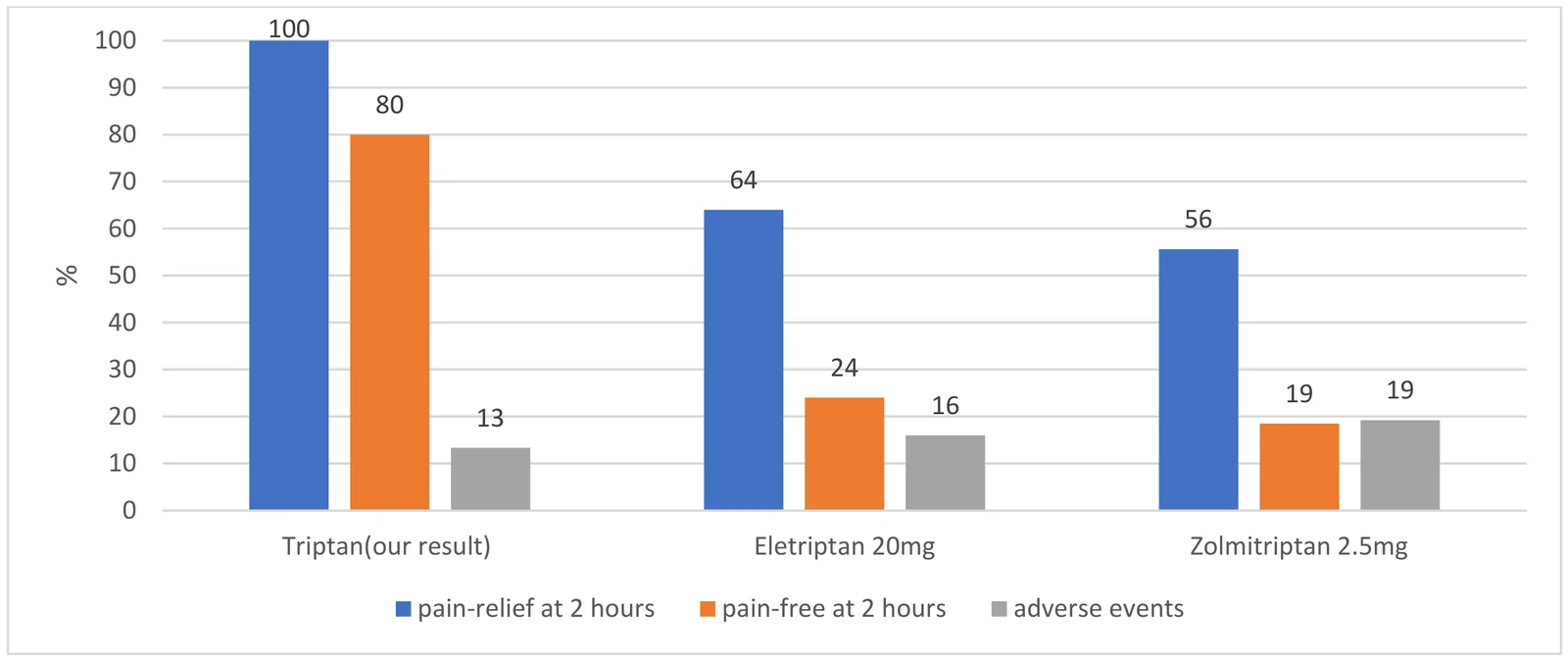
Pain-free and pain-relief rates of triptans indirectly compared with reported clinical trial data in Japan.

**Figure 2:**
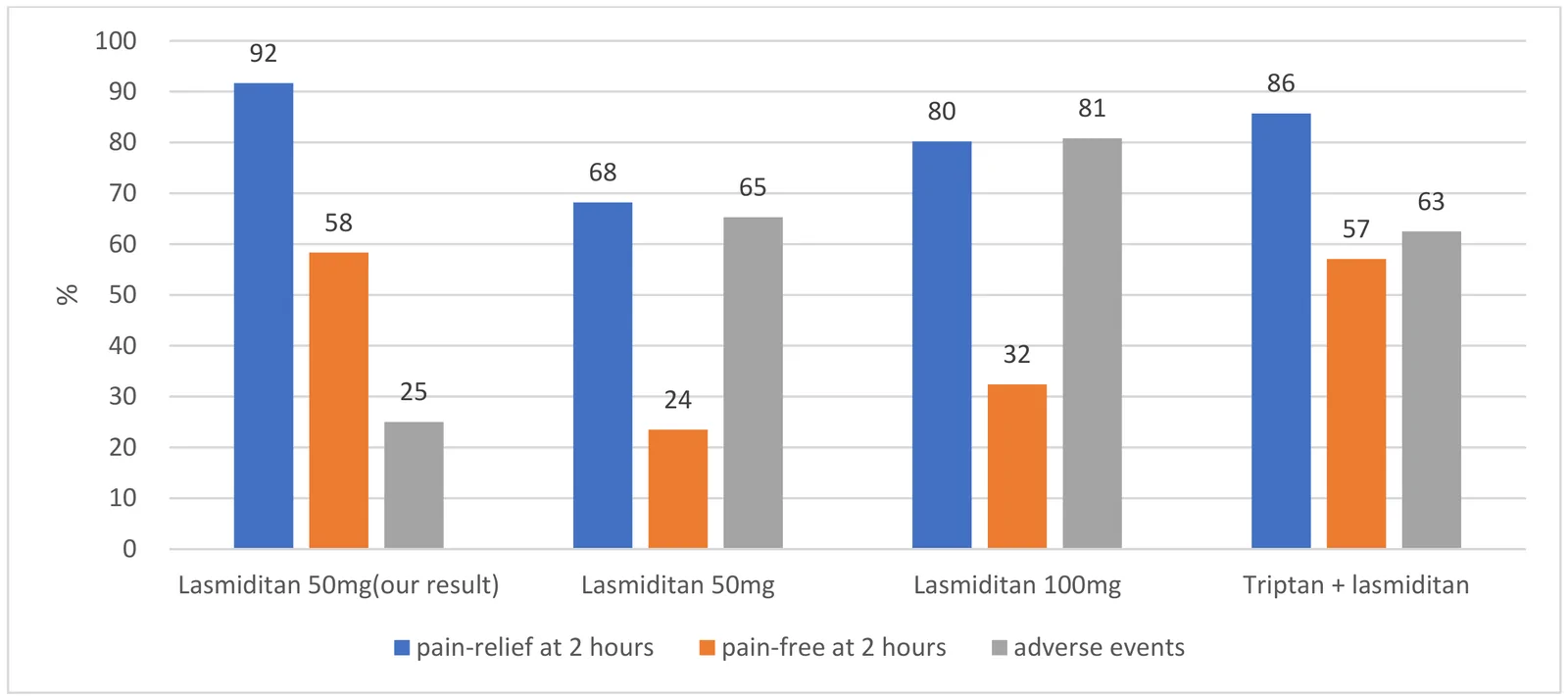
Pain-free and pain-relief rates of lasmiditan indirectly compared with the reported clinical trial data in Japan.

**Table 1:** Blank paper headache diary.

Date	Menstruation	Headache				Disability	Triger	Other Symptoms	Adverse Effects
		☐	Morning	Afternoon	Night				
☐	☐	severity	☐	☐	☐	☐	☐	☐	☐
		medication	☐	☐	☐				
☐	☐	severity	☐	☐	☐	☐	☐	☐	☐
		medication	☐	☐	☐				
☐	☐	severity	☐	☐	☐	☐	☐	☐	☐
		medication	☐	☐	☐				

**Table 2:** Characteristics of the study population.

Age	Sex	Migraine	Preventive medication	Neurodevelopmental Disorder	Triptan			Lasmiditan 50	
					Brand	Pain Relief /Pain-Free	Adverse Event	Pain Relief /Pain-Free	Adverse Event
18	F	CM, MoA	Lomerizine	ASD	Eletriptan	+/+	−	−/−	Mild somnolence
12	M	CM, MwA	Lomerizine	ASD	Zolmitriptan	+/+	Throat discomfort	+/+	Mild somnolence
15	M	EM, MoA	None	ADHD	Zolmitriptan	+/−	Throat discomfort	+/−	−
30	F	CM, MoA	Lomerizine, fremanezumab	ASD	Zolmitriptan	+/+	−	+/+	−
39	M	EM, MoA	Lomerizine	Asperger’s syndrome	☐N/A	N/A	N/A	+/−	−
14	F	CM, MwA	Lomerizine	ADHD	☐N/A	N/A	N/A	+/−	−
36	M	EM, MoA	Lomerizine	ADHD	Eletriptan	+/+	−	+/+	−
29	F	CM, MwA	Lomerizine	Asperger’s syndrome	Eletriptan	+/+	−	+/+	−
39	F	CM, MoA	Lomerizine, galcanezumab	ASD	Eletriptan	+/−	−	+/−	−
10	M	EM, MwA	None	ADHD	Zolmitriptan	+/−	−	N/A	N/A
13	F	CM, MoA	Amitriptyline	ASD	Eletriptan	+/+	−	N/A	N/A
23	F	EM, MoA	Lomerizine	ADHD	Eletriptan	+/+	−	+/+	−
9	M	EM, MoA	None	ADHD	Eletriptan	+/+	−	N/A	N/A
17	M	EM, MoA	lomerizine	ASD	Eletriptan	+/+	−	N/A	N/A
28	F	CM, MoA	lomerizine fremanezumab	ASD	Eletriptan	+/+	−	N/A	N/A
30	F	CM, MwA	lomerizine	ADHD	Eletriptan	+/+	−	+/+	Mild dizziness
17	M	EM, MoA	lomerizine	ADHD	Eletriptan	+/+	−	+/+	−

M: male, F: female, MoA: migraine without aura, MwA: migraine with aura, ASD: autism spectrum disorder, ADHD: attention-deficit hyperactive disorder, N/A: not available, −: not observed, +/+: achieved both pain relief and pain-free, +/−: achieved only pain relief, −/−: did not achieve pain relief.

**Table 3:** Summary of patient characteristics.

Age	Years	9–39
sex	male/female	8/9
Migraine phenotype	with/without aura	5/12
	episodic/chronic	8/9
preventive medication	none	3
	lomerizine	13
	amitriptyline	1
	galcanezumab	1
	fremanezumab	1
neurodevelopmental disorder diagnosis	ASD	7
	Asperger’s syndrome	2
	ADHD	8
response to triptan	pain relief	15/15
	pain free	12/15
	adverse events	2/15
response to lasmiditan	pain relief	11/12
	pain free	7/12
	adverse events	3/12

ASD: autism spectrum disorder, ADHD: attention-deficit hyperactive disorder.

## Data Availability

The original contributions presented in this study are included in the article.

## Abbreviations

The following abbreviations are used in this manuscript:

ADHD	Attention-deficit/hyperactivity disorder
ASD	Autism spectrum disorder
CGRP	Calcitonin gene-related peptide
DSM	Diagnostic and Statistical Manual of Mental Disorders
5-HT	Serotonin
M	Male
F	Female
MoA	Migraine without aura
MwA	Migraine with aura
NSAIDs	Nonsteroidal anti-inflammatory drugs

## References

[1] Ashina M., Katsarava Z., Do T.P., Buse D.C., Pozo-Rosich P., Özge A., Krymchantowski A.V., Lebedeva E.R., Ravishankar K., Yu S. (2021). Migraine: Epidemiology and systems of care. Lancet.

[2] Saylor D., Steiner T. (2018). The Global Burden of Headache. Semin. Neurol..

[3] GBD 2016 Headache Collaborators (2018). Global, regional, and national burden of migraine and tension-type headache, 1990–2016: A systematic analysis for the Global Burden of Disease Study 2016. Lancet Neurol..

[4] Ljubisavljevic S., Ljubisavljevic M., Damjanovic R., Kalinic S. (2023). A Descriptive Review of Medication-Overuse Headache: From Pathophysiology to the Comorbidities. Brain Sci..

[5] Goadsby P.J., Lipton R.B., Ferrari M.D. (2002). Migraine–current understanding and treatment. N. Engl. J. Med..

[6] Lassen L.H., Haderslev P.A., Jacobsen V.B., Iversen H.K., Sperling B., Olesen J. (2002). Cgrp May Play A Causative Role in Migraine. Cephalalgia.

[7] Russell F.A., King R., Smillie S.-J., Kodji X., Brain S.D. (2014). Calcitonin Gene-Related Peptide: Physiology and Pathophysiology. Physiol. Rev..

[8] Croop R., Goadsby P.J., Stock D.A., Conway C.M., Forshaw M., Stock E.G., Coric V., Lipton R.B. (2019). Efficacy, safety, and tolerability of rimegepant orally disintegrating tablet for the acute treatment of migraine: A randomised, phase 3, double-blind, placebo-controlled trial. Lancet.

[9] Goadsby P.J., Dodick D.W., Ailani J., Trugman J.M., Finnegan M., Lu K., Szegedi A. (2020). Safety, tolerability, and efficacy of orally administered atogepant for the prevention of episodic migraine in adults: A double-blind, randomised phase 2b/3 trial. Lancet Neurol..

[10] Clemow D.B., Johnson K.W., Hochstetler H.M., Ossipov M.H., Hake A.M., Blumenfeld A.M. (2020). Lasmiditan mechanism of action—Review of a selective 5-HT1F agonist. J. Headache Pain.

[11] Tepper S.J., Vasudeva R., Krege J.H., Rathmann S.S., Doty E., Vargas B.B., Magis D., Komori M. (2020). Evaluation of 2-Hour Post-Dose Efficacy of Lasmiditan for the Acute Treatment of Difficult-to-Treat Migraine Attacks. Headache J. Head Face Pain.

[12] Genizi J., Gordon S., Kerem N.C., Srugo I., Shahar E., Ravid S. (2013). Primary headaches, attention deficit disorder and learning disabilities in children and adolescents. J. Headache Pain.

[13] Association A.P. (2025). Diagnostic and Statistical Manual of Mental Disorders.

[14] Nwaobi S.E. (2026). Useful considerations for treating migraine in patients with autism. Headache J. Head Face Pain.

[15] Grant Tejada M.P., Klomhaus A.M., Ortiz R., Tibbe T.D., Nwaobi S.E. (2025). Migraine prevalence and phenotype in autism: A retrospective cohort study using a US National Health Survey and large academic health system electronic health record. Headache J. Head Face Pain.

[16] Alabaf S., Gillberg C., Lundström S., Lichtenstein P., Kerekes N., Råstam M., Anckarsäter H. (2019). Physical health in children with neurodevelopmental disorders. J. Autism Dev. Disord..

[17] Hollander E., Novotny S., Allen A., Aronowitz B., Cartwright C., DeCaria C. (2000). The Relationship between Repetitive Behaviors and Growth Hormone Response to Sumatriptan Challenge in Adult Autistic Disorder. Neuropsychopharmacology.

[18] Faraone S.V., Ward C.L., Boucher M., Elbekai R., Brunner E. (2025). Role of serotonin in the neurobiology of attention-deficit/hyperactivity disorder: A systematic literature review. Expert Opin. Ther. Targets.

[19] Murphy D.G.M., Daly E., Schmitz N., Toal F., Murphy K., Curran S., Erlandsson K., Eersels J., Kerwin R., Ell P. (2006). Cortical Serotonin 5-HT 2A Receptor Binding and Social Communication in Adults With Asperger’s Syndrome: An in Vivo SPECT Study. Am. J. Psychiatry.

[20] Makkonen I., Riikonen R., Kokki H., Airaksinen M.M., Kuikka J.T. (2008). Serotonin and dopamine transporter binding in children with autism determined by SPECT. Dev. Med. Child Neurol..

[21] Headache Classification Committee of the International Headache Society (IHS) (2018). The International Classification of Headache Disorders, 3rd edition. Cephalalgia.

[22] Sakai F., Takeshima T., Homma G., Tanji Y., Katagiri H., Komori M. (2021). Phase 2 randomized placebo-controlled study of lasmiditan for the acute treatment of migraine in Japanese patients. Headache J. Head Face Pain.

[23] Marmura M.J., Silberstein S.D., Schwedt T.J. (2015). The Acute Treatment of Migraine in Adults: The American Headache Society Evidence Assessment of Migraine Pharmacotherapies. Headache J. Head Face Pain.

[24] Puledda F., Sacco S., Diener H.-C., Ashina M., Al-Khazali H.M., Ashina S., Burstein R., Liebler E., Cipriani A., Chu M.K. (2024). International Headache Society global practice recommendations for the acute pharmacological treatment of migraine. Cephalalgia.

[25] Peles I., Shneyour R.S., Levanon E., Steen Y.M., Abu Salameh I., Gordon M., Abuhasira R., Novack V., Ifergane G. (2025). Cardiovascular risk and triptan usage among patients with migraine. Headache J. Head Face Pain.

[26] Ashina M., Buse D.C., Ashina H., Pozo-Rosich P., Peres M.F.P., Lee M.J., Terwindt G.M., Singh R.H., Tassorelli C., Do T.P. (2021). Migraine: Integrated approaches to clinical management and emerging treatments. Lancet.

[27] Dodick D.W., Lipton R.B., Ailani J., Halker Singh R.B., Shewale A.R., Zhao S., Trugman J.M., Yu S.Y., Viswanathan H.N. (2020). Ubrogepant, an Acute Treatment for Migraine, Improved Patient-Reported Functional Disability and Satisfaction in 2 Single-Attack Phase 3 Randomized Trials, ACHIEVE I and II. Headache J. Head Face Pain.

[28] Lipton R.B., Blumenfeld A., Jensen C.M., Croop R., Thiry A., L’Italien G., Morris B.A., Coric V., Goadsby P.J. (2023). Efficacy of rimegepant for the acute treatment of migraine based on triptan treatment experience: Pooled results from three phase 3 randomized clinical trials. Cephalalgia.

[29] Polavieja P., Belger M., Venkata S.K., Wilhelm S., Johansson E. (2022). Relative efficacy of lasmiditan versus rimegepant and ubrogepant as acute treatments for migraine: Network meta-analysis findings. J. Headache Pain.

[30] Manganotti P., Deodato M., D’Acunto L., Biaduzzini F., Garascia G., Granato A. (2024). Effects of Anti-CGRP Monoclonal Antibodies on Neurophysiological and Clinical Outcomes: A Combined Transcranial Magnetic Stimulation and Algometer Study. Neurol. Int..

[31] Oliveira R., Gil-Gouveia R., Puledda F. (2024). CGRP-targeted medication in chronic migraine—Systematic review. J. Headache Pain.

[32] Mathew N.T., Tietjen G.E., Lucker C. (1996). Serotonin syndrome complicating migraine pharmacotherapy. Cephalalgia.

[33] Evans R.W., Tepper S.J., Shapiro R.E., Sun-Edelstein C., Tietjen G.E. (2010). The FDA Alert on Serotonin Syndrome With Use of Triptans Combined With Selective Serotonin Reuptake Inhibitors or Selective Serotonin-Norepinephrine Reuptake Inhibitors: American Headache Society Position Paper. Headache J. Head Face Pain.

[34] Boylan C.B., Blue M.E., Hohmann C.F. (2007). Modeling early cortical serotonergic deficits in autism. Behav. Brain Res..

[35] Chugani D.C. (2004). Serotonin in autism and pediatric epilepsies. Ment. Retard. Dev. Disabil. Res. Rev..

[36] Nakamura K., Sekine Y., Ouchi Y., Tsujii M., Yoshikawa E., Futatsubashi M., Tsuchiya K.J., Sugihara G., Iwata Y., Suzuki K. (2010). Brain Serotonin and Dopamine Transporter Bindings in Adults With High-Functioning Autism. Arch. Gen. Psychiatry.

[37] Fukuuchi Y. (2002). Efficacy and Safety of Eletriptan 20 Mg, 40 Mg and 80 Mg in Japanese Migraineurs. Cephalalgia.

[38] Sakai F., Iwata M., Tashiro K., Itoyama Y., Tsuji S., Fukuuchi Y., Sobue G., Nakashima K., Morimatsu M. (2002). Zolmitriptan is Effective and Well Tolerated in Japanese Patients with Migraine: A Dose-Response Study. Cephalalgia.

[39] Shibata Y., Sato H., Sato A., Harada Y. (2024). Efficacy of Lasmiditan as a Secondary Treatment for Migraine Attacks after Unsuccessful Treatment with a Triptan. Neurol. Int..

[40] Katsuki M., Yamada Y., Ichihara T., Matsumori Y. (2025). Time-series clustering analysis for treatment pattern of lasmiditan for 2 years from the initial prescription. Ther. Adv. Neurol. Disord..

[41] Tassorelli C., Bragg S., Krege J.H., Doty E.G., Ardayfio P.A., Ruff D., Dowsett S., Schwedt T. (2021). Safety findings from CENTURION, a phase 3 consistency study of lasmiditan for the acute treatment of migraine. J. Headache Pain.

[42] Goadsby P.J., Wietecha L.A., Dennehy E.B., Kuca B., Case M.G., Aurora S.K., Gaul C. (2019). Phase 3 randomized, placebo-controlled, double-blind study of lasmiditan for acute treatment of migraine. Brain.

[43] Srikiatkhachorn A., Anthony M. (1996). Platelet Serotonin in Patients with Analgesic-Induced Headache. Cephalalgia.

[44] Srikiatkhachorn A., Anthony M. (1996). Serotonin Receptor Adaptation in Patients with Analgesic-Induced Headache. Cephalalgia.

[45] Ashina M., Reuter U., Smith T., Krikke-Workel J., Klise S.R., Bragg S., Doty E.G., Dowsett S.A., Lin Q., Krege J.H. (2021). Randomized, controlled trial of lasmiditan over four migraine attacks: Findings from the CENTURION study. Cephalalgia.

